# Supplementary material for: Synthesis of Deuterium-Labeled Vitamin D Metabolites as Internal Standards for LC-MS Analysis
Source: Molecules. 2022 Apr 9;27(8):2427. doi: 10.3390/molecules27082427 (PMC9025501; doi:10.3390/molecules27082427)
Supplement: Supplementary file 1 [file molecules-27-02427-s001.zip › molecules-1665794-supplementary.pdf]

molecules

Supporting Information

Synthesis of Deuterium-Labeled Vitamin D Metabolites as Internal Standards for LC-MS Analysis

Akiko Nagata [1], Kazuto Iijima [1], Ryota Sakamoto [1], Yuka Mizumoto [1], Miho Iwaki [1], Masaki Takiwaki [2], Yoshikuni Kikutani [2], Seketsu Fukuzawa [2], Minami Odagi [1], Masayuki Tera [1] and Kazuo Nagasawa [1,*]

[1] Department of Biotechnology and Life Science, Tokyo University of Agriculture and Technology, Koganei, Tokyo 184-8588, Japan; s193819x@st.go.tuat.ac.jp (A.N.); s206189q@st.go.tuat.ac.jp (K.I.); s218969x@st.go.tuat.ac.jp (R.S.); s200343y@st.go.tuat.ac.jp (Y.M.); s218363r@st.go.tuat.ac.jp (M.I.); odagi@cc.tuat.ac.jp (M.O.); tera@go.tuat.ac.jp (M.T.)

[2] Medical Equipment Business Operations, Management Strategy Planning Division, JEOL Ltd., Akishima, Tokyo 196-8558, Japan; mtakiwak@jeol.co.jp (M.T.); ykikutan@jeol.co.jp (Y.K.); sfukuzaw@jeol.co.jp (S.F.)

* Correspondence: knaga@cc.tuat.ac.jp; Tel.: + 81-42-388-7295

Table of Contents

1) Experimental procedures for synthesis and characterization of compounds

1.1 General S2

1.2 Synthesis of deuterium-labeled vitamin D_3 metabolites **2**, **6**, and **7** S3-S11

2) Experimental procedure for LC-MS/MS analysis using the isotope dilution method

2.1 Materials S12

2.2 Preparation of standard samples (DAP-PA-derivatized vitamin D metabolites) S12

2.3 Optimization of LC MS/MS conditions S12-S13

2.4 Preparation of calibrators and internal standard solutions: S13

3) ^{1}H and ^{13}C NMR spectra S14-S23

1. Experimental procedures for synthesis and characterization of compounds

1.1 General

Unless otherwise stated, reactions were performed under an argon atmosphere using freshly dried solvents. All reactions were monitored by thin-layer chromatography using Merck silica gel 60 F_{254} pre-coated plates (0.25 mm) and were visualized by UV, *p*-anisaldehyde staining. Flash column chromatography was performed under pressurization using silica gel (particle size 40-100 μm) purchased from Kanto Chemical. High-pressure liquid chromatography (HPLC) purification was performed with a Hitachi instrument with GL Scieneces InterSustain PFP column (4.6X250 mm). Optical rotations were measured on a JASCO P-2200 polarimeter. ^{1}H and ^{13}C NMR spectra were recorded on JEOL JNM-AL300 (300 MHz), JEOL JNM-ECX 400 (400 MHz) and JEOL JNM-ECA 500 (500 MHz). The spectra are referenced internally according to residual solvent signals of $CDCl_3$ (^{1}H NMR; δ = 7.26 ppm, ^{13}C NMR; δ= 77.0 ppm). Data for ^{1}H NMR are recorded as follows: chemical shift (δ, ppm), multiplicity (s, singlet; d, doublet; t, triplet; q, quartet; m, multiplet; br, broad), coupling constant (Hz), integration. Data for ^{13}C NMR are reported in terms of chemical shift (δ, ppm). Mass spectra were recorded on JEOL JMS-T100X mass with ESI-MS mode using methanol as solvent.

1.2 Synthesis of deuterium-labeled vitamin D_3 metabolites **2**, **6**, and **7**

3,4-dihydroxybutyl-3,4,4-*d*3 pivalate (**10**-*d*3).

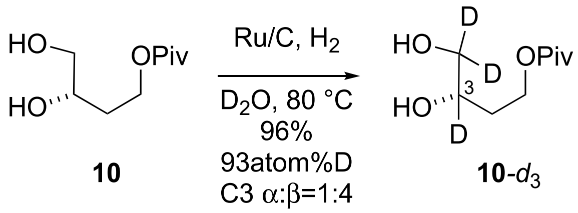

A suspension of triol **10** (4.0 g 21.0 mmol) and 5% Ru/C (4.0g 5 mol%) in D_2O (80 mL) was stirred at 80 °C under a hydrogen atmosphere for 24h. After the reaction, the mixture was cooled to room temperature and filtered through a pad of celite, and the filtrates were concentrated *in vacuo*. The residue was purified by column chromatography on silica gel (*n*-hexane/ethyl acetate = 1:1) to give **10**-*d*3 (3.84 g, 96%) as a colorless oil. Spectral data for **10**-*d*3: $[\alpha]^{25}_{D}$= -5.7 (*c* 2.1, $CHCl_3$); ^{1}H NMR (400 MHz, $CDCl_3$) δ 4.34-4.42 (m, 1H), 4.12-4.17 (m, 1H), 1.68-1.81 (m, 2H), 1.19 (s, 9H) ppm; ^{13}C NMR (100 MHz, $CDCl_3$) δ 178.9, 68.6, 68.4, 68.1, 65.7, 65.5, 61.1, 38.58, 31.9, 27.0 ppm; HRMS (ESI, M+Na) calcd. for $C_9H_{15}D_3O_4Na$ 216.1291, found 216.1268.

3-hydroxy-6-(trimethylsilyl)hex-5-yn-1-yl-3,4,4-*d*3 pivalate (**11**).

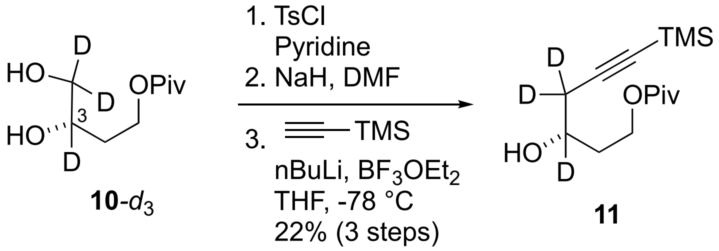

To a solution of **10**-*d*3 (7.8 g 40.4 mmol) in pyridine (80 mL) was added *p*-toluene sulfonyl chloride (1.29 g, 11.0 mmol) at room temperature, and the reaction mixture was stirred at the same temperature for 15 h. The reaction mixture was quenched with H_2O (80 mL) and the mixture was extracted with dichloromethane three times. The combined organic layer was washed with brine, dried over $MgSO_4$, filtered, and concentrated *in vacuo*. The residue was purified by column chromatography on silica gel (*n*-hexane/ethyl acetate = 3:1) to give tosyl-*d*3 (7.05 g, 50%). To a solution of tosyl-*d*3 (10.8 g, 31.1 mmol) in dimethylformamide (156 mL) was added NaH (1.49 g, 37.3 mmol) at 0 °C, and the resulting mixture was stirred at room temperature for 0.5 h. The reaction mixture was quenched with H_2O (100 mL) and the mixture was extracted with dichloromethane three times. The combined organic layer was washed with brine, dried over $MgSO_4$, filtered, and concentrated *in vacuo*. The residue was purified by column chromatography on silica gel (*n*-hexane/ethyl acetate = 8:1) to give epoxide-*d*3 (3.96 g, 72%). To a solution of trimethylsilyl acetylene (6.4 mL, 45.6 mmol) in THF (25 mL) was dropwise added *n*-BuLi (1.6M in hexane, 13.7 mL, 35.6 mmol) at -78 °C. After stirred for 1h at the same temperature, the solution of epoxide-*d*3 (3.47 g 19.8 mmol) in THF (16.5 mL) and BF_3 Et_2O (3.23 mL, 25.7 mmol) was added to the reaction mixture at -78 °C. After stirred for 0.5 h at the same temperature, the reaction was quenched with sat. NH_4Claq. (20 mL) and the mixture was extracted with ethyl acetate three times. The combined organic layer was washed with brine, dried over $MgSO_4$, filtered, and concentrated *in vacuo*. The residue was purified by column chromatography on silica gel (*n*-hexane/ethyl acetate = 10:1) to give **11** (3.33 g, 61%) as a colorless oil. Spectral data for **11**: $[\alpha]^{25}_{D}$= -2.2 (*c* 1.3, $CHCl_3$); ^{1}H NMR (300 MHz, $CDCl_3$) δ 4.27-4.33 (m, 1H), 4.15-4.21 (m, 1H), 1.88-1.95 (m, 1H), 1.75-1.81 (m, 1H), 1.18 (s, 9H), 0.15 (s, 9H) ppm; ^{13}C NMR (100 MHz, $CDCl_3$) δ 179.8, 76.4, 73.6, 69.1, 56.5, 52.4, 43.3, 41.8, 41.4, 40.2, 33.4, 32.9, 27.4, 24.2, 22.4, 18.9, 17.3, 13.4 ppm; HRMS (ESI, M+Na) calcd. for $C_{14}H_{23}D_3O_3SiNa$ 296.1737, found 296.1732.

3-((*tert*-butyldimethylsilyl)oxy)hex-5-yn-3,4,4-*d*$_3$-1-ol (12).

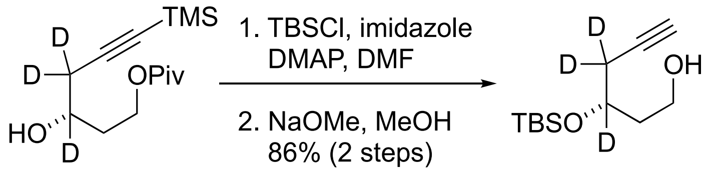

11 **12**

To a solution of **11** (3,2 g, 11.7 mmol) in dimethylformamide (23.4 mL) was added imidazole (2.15 g, 31.6 mmol), tert-butyldimethylchlorosilane (3.5 g, 23 mmol), 4-dimethyl amino pyridine (143 mg, 1.17 mmol) at room temperature, and the resulting mixture was stirred at room temperature for 3 h. The reaction mixture was quenched with H_2O (25 mL) and the mixture was extracted with a solution of *n*-hexane/ethyl acetate = 4:1 three times. The combined organic layer was washed with brine, dried over $MgSO_4$, filtered, and concentrated *in vacuo*. The residue was purified by column chromatography on silica gel (*n*-hexane/ethyl acetate = 10:1) to give alkyne-*d*$_3$ (4.61 g, 100%). To a solution of alkyne-*d*$_3$ (4,51 g, 11.6 mmol) in MeOH (107 mL) was added NaOMe (5 M solution in MeOH, 9.28 mL, 46.4 mmol) at 0 °C, and the mixture was stirred at the room temperature for 24 h. The reaction mixture was quenched with saturated NH_4Cl aq., and the mixture was extracted with ethyl acetate three times. The combined organic layer was washed with brine, dried over $MgSO_4$, filtered, and concentrated *in vacuo*. The residue was purified by column chromatography on silica gel (*n*-hexane/ethyl acetate = 30:1) to give **12** (2.32 g, 86%) as a colorless oil. Spectral data for **12**: $[\alpha]^{25}{}_{D}$= -16.4 (*c* 1.3, $CHCl_3$); ^{1}H NMR (300 MHz, $CDCl_3$) δ 3.79-3.85 (m, 1H), 3.71-3.77 (m, 1H), 1.99 (s, 1H), 1.91-1.97 (m, 1H), 1.77-1.84 (m, 1H), 0.89 (s, 9H), 0.11 (s, 6H) ppm; ^{13}C NMR (100 MHz, $CDCl_3$) δ 80.9, 70.4, 69.4, 69.1, 59.7, 37.6, 26.6, 25.7, 17.9, -4.6, -4.9 ppm; HRMS (ESI, M+Na) calcd. for $C_{12}H_{21}D_3O_2SiNa$ 254.1632, found 254.1631.

***tert*-butyldimethyl((oct-7-en-1-yn-4-yl-3,3,4-*d*$_3$)oxy)silane (13).**

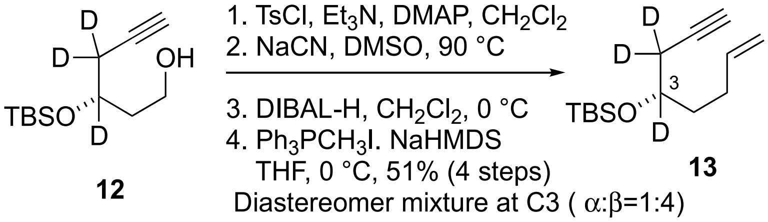

To a solution of **12** (1.0 g, 4.32 mmol) in CH_2Cl_2 (14 mL) was added Et_3N (2.1 mL, 15.1 mmol), *p*-toluene sulfonyl chloride (1.29 g, 11.0 mmol), 4-dimethyl amino pyridine (143 mg, 1.17 mmol) at room temperature, and the reaction mixture was stirred at the same temperature for 2 h. The reaction mixture was quenched with H_2O (20 mL) and the mixture was extracted with dichloromethane three times. The combined organic layer was washed with brine, dried over $MgSO_4$, filtered, and concentrated *in vacuo*. The residue was purified by column chromatography on silica gel (*n*-hexane/ethyl acetate = 20:1) to give tosyl-d$_3$ (1.67 g, 99%). To a solution of tosyl-*d*$_3$ (1.67 g, 4.27 mmol) in DMSO (14 mL) was added NaCN (314 mg, 6.41 mmol), at room temperature, and the reaction mixture was stirred at the 90 °C for 0.5 h. The reaction mixture was quenched with H_2O (20 mL) and the mixture was extracted with dichloromethane three times. The combined organic layer was washed with brine, dried over $MgSO_4$, filtered, and concentrated *in vacuo*. The residue was purified by column chromatography on silica gel (*n*-hexane/ethyl acetate = 20:1) to give cyanide (1.0 g, 100%). To a solution of cyanide (505.9 mg, 2.09 mol) in CH_2Cl_2 (5.6 mL) were added DIBAL-H (1 M solution in hexane, 2.5 mL, 2.5 mmol) in sequence at 0 °C, and the reaction mixture was stirred at the same temperature for 1 h. To the reaction mixture was added 2-propanol (1.75 mL) at 0 °C, and the

resultant mixture was warmed to room temperature. Then, H_2O and silica gel were added, and allowed to stir for 0.5 h. The slurry was filtered through a pad of celite, and the filtrates were concentrated *in vacuo*. The crude residue was used in the next step without further purification. To a stirred solution of methyltriphenylphosphonium iodide (2.79 g, 6.9 mmol) in THF (15 mL) was added NaHMDS (1.9 M solution in THF, 3.2 mL, 6.15 mmol) at 0 °C. After stirred for 30 min at the same temperature, a solution of crude residue in THF (9.0 mL) was added to the reaction mixture at 0 °C. After stirred for 1 h at the same temperature, the reaction was quenched with H_2O (20 mL) and the mixture was extracted with ethyl acetate three times. The combined organic layer was washed with brine, dried over $MgSO_4$, filtered, and concentrated *in vacuo*. The residue was purified by column chromatography on silica gel (*n*-hexane/ethyl acetate = 100:1) to give **13** (461.2 mg, 61% 2 steps) as a colorless oil. Spectral data for **13**: $[\alpha]^{25}_{D}$= -10.0 (*c* 1.0, $CHCl_3$); ^1H NMR (300 MHz, $CDCl_3$) δ 5.89-5.76 (m, 1H), 5.03 (dd, *J* = 16.9, 1.8 Hz, 1H), 4.96 (d, *J* = 10.1 Hz, 1H), 2.21-2.00 (m, 2H), 1.97 (s, 1H), 1.79-1.66 (m, 1H), 1.66-1.58 (m, 1H), 0.89 (s, 9H), 0.07 (d, *J* = 4.6 Hz, 6H) ppm; ^{13}C NMR (100 MHz, $CDCl_3$) δ 138.5, 114.5, 81.4, 70.2, 70.0, 69.8, 69.6, 35.5, 29.3, 26.9, 26.6, 25.8, 18.1, -4.5, -4.7 ppm; HRMS (ESI, M+Na) was obtained as deprotected form of alcohol, calcd. for $C_8H_9D_3ONa$ 150.0974, found 150.0980.

(*E*)-5-((*tert*-butyldimethylsilyl)oxy)oct-2-en-7-yn-5,6,6-*d*3-1-ol (**14**).

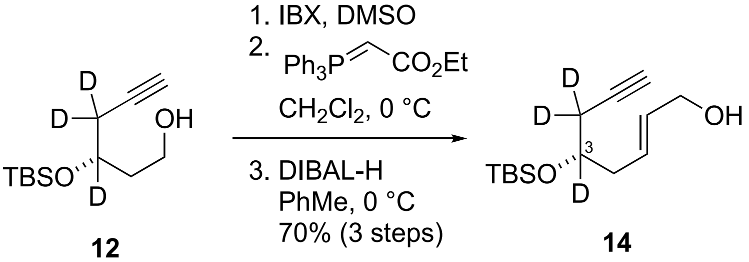

12 **14**

To a solution of **12** (0.80 g, 3.46 mmol) in DMSO (35 mL) was added 2-iodoxybenzoic acid (1.93 g, 6.91 mmol), at room temperature, and the reaction mixture was stirred at the 50 °C for 0.5 h. The reaction mixture was quenched with sat. $Na_2S_2O_3$ aq. (35 mL) and the mixture was extracted with ethyl acetate three times. The combined organic layer was washed with brine, dried over $MgSO_4$, filtered, and concentrated *in vacuo*. The residue was purified by column chromatography on silica gel (*n*-hexane/ethyl acetate = 50:1) to give aldehyde-*d*3 (0.57 g, 72%). To a solution of aldehyde-*d*3 (0.55 g, 2.41 mmol) in dichloromethane (24 mL) was added ethyl (triphenylphosphoranylidene)acetate (1.68 g, 4.83 mmol) at 0 °C, and the reaction mixture was stirred at the same temperature for 1 h. The reaction mixture was quenched with H_2O (24 mL) and the mixture was extracted with dichloromethane three times. The combined organic layer was washed with brine, dried over $MgSO_4$, filtered, and concentrated *in vacuo*. The residue was purified by column chromatography on silica gel (*n*-hexane/ethyl acetate = 100:1) to give ethyl ester-*d*3 (0.72 g, 99%). To a solution of ethyl ester-d3 (0.72 g, 2.41 mol) in dichloromethane (24.1 mL) were added DIBAL-H (1 M solution in hexane, 7.2 mL, 7.2 mmol) in sequence at 0 °C, and the reaction mixture was stirred at the same temperature for 0.5 h. To the reaction mixture was added 2-propanol (5.0 mL) at 0 °C, and the resultant mixture was warmed to room temperature. Then, H_2O and silica gel were added, and allowed to stir for 0.5 h. The slurry was filtered through a pad of celite, and the filtrates were concentrated *in vacuo*. The residue was purified by column chromatography on silica gel (*n*-hexane/ethyl acetate = 10:1) to give **14** (0.60 g, 98%) as a colorless oil. Spectral data for **14**: $[\alpha]^{25}_{D}$= -2.7 (*c* 0.8, $CHCl_3$); ^1H NMR (300 MHz, $CDCl_3$) δ 4.11 (d, *J* = 4.1 Hz, 2H), 2.41-2.37 (m, 1H), 2.28 (dd, *J* = 12.6, 4.4 Hz, 1H), 1.98 (s, 1H), 0.89 (s, 9H), 0.07 (d, *J* = 6.9 Hz, 6H) ppm; ^{13}C NMR (100 MHz, $CDCl_3$) δ 132.2, 128.5, 81.5, 70.4, 70.2, 70.0, 63.7, 39.3, 26.5, 25.9, 18.2, -4.5, -4.6 ppm; HRMS (ESI, M+Na) calcd. for $C_{14}H_{23}D_3O_2SiNa$ 280.1788, found 280.1791.

5-((*tert*-butyldimethylsilyl)oxy)oct-1-en-7-yn-5,6,6-*d*$_3$-3-ol (**15**).

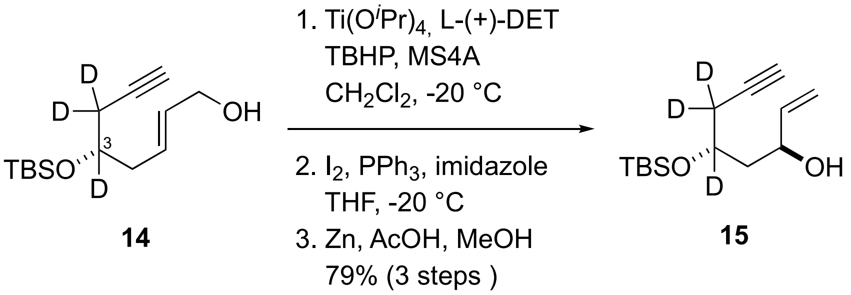

To a mixture of dried MS 4A (1.18 g) in CH_2Cl_2 (18 mL) was added Ti(O^{i}Pr)$_4$ (0.21 mL, 0.70 mmol), L-(+)-DET (0.15 mL, 0.85 mmol), a solution of **14** (0.60 g, 2.35 mmol) in CH_2Cl_2 (6 mL), TBHP (3.5 M solution in dichloromethane, 1.34 mL, 4.7 mmol) at -20 °C, and the reaction mixture was stirred at the same temperature for 1 h. The reaction mixture was quenched with SMe_2 (2.5 mL) and sat. NaF aq. (70 mL) and allowed to stir for 0.5 h. The mixture was extracted with dichloromethane three times. The combined organic layer was washed with brine, dried over $MgSO_4$, filtered, and concentrated *in vacuo*. The residue was purified by column chromatography on silica gel (*n*-hexane/ethyl acetate = 20:1) to give epoxide-*d*$_3$ (0.58 g, 91%). To a solution of epoxide-*d*$_3$ (0.51 g, 1.85 mmol) in THF (9 mL) was added triphenylphosphine (1.46 g, 5.6 mmol), imidazole (755.7 mg, 11.1 mmol), iodide (1.41 g, 5.55 mmol) at -20 °C, and the reaction mixture was stirred at the same temperature for 0.5 h. The reaction mixture was quenched with sat. $Na_2S_2O_3$ aq. (9 mL) and the mixture was extracted with ethyl acetate three times. The combined organic layer was washed with brine, dried over $MgSO_4$, filtered, and concentrated *in vacuo*. The residue was purified by column chromatography on silica gel (*n*-hexane/ethyl acetate = 80:1) to give iodide-*d*$_3$ (0.62 g, 88%). To a solution of iodide-*d*$_3$ (0.58 g, 1.51 mmol) in MeOH (15 mL) was added acetic acid (0.58 mL), Zn powder (345.9mg, 5.29 mmol), at room temperature, and the reaction mixture was stirred at the same temperature for 1 h. The reaction mixture was filtered through a pad of celite, and the filtrates were concentrated *in vacuo*. The residue was purified by column chromatography on silica gel (*n*-hexane/ethyl acetate = 80:1) to give **15** (0.62 g, 88%) as a colorless oil. Spectral data for **15**: HRMS (ESI, M+Na) calcd. for $C_{14}H_{23}D_3O_2SiNa$ 280.1788, found 280.1804.

(3*S*)-5-((*tert*-butyldimethylsilyl)oxy)oct-1-en-7-yn-5,6,6-*d*$_3$-3-ol ((-)-**15**).

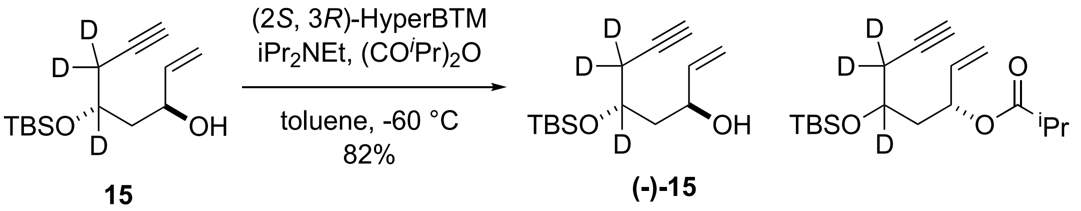

To a solution of **15** (332.7 mg, 1.29 mmol) in toluene (26 mL) was added (2*S*,3*R*)-HyperBTM (40.1 mg, 0.13 mmol, 10 mol%), iPr$_2$NEt (68 μL), (CO^{i}Pr)$_2$O (65 μL) at -60 °C, and the reaction mixture was stirred at the same temperature for 8 h. The reaction mixture was quenched with H_2O (20 mL) and the mixture was extracted with ethyl acetate three times. The combined organic layer was washed with brine, dried over $MgSO_4$, filtered, and concentrated *in vacuo*. The residue was purified by column chromatography on silica gel (*n*-hexane/ethyl acetate = 100:1) to give (-)-**15** (269.7 mg, 82%) as a colorless oil. Spectral data for (-)-**15**: $[\alpha]^{25}_{D}$= -15.5 (*c* 0.6, $CHCl_3$); ^1H NMR (400 MHz, $CDCl_3$) δ 5.92-5.82 (m, 1H), 5.30-5.24 (m, 1H), 5.10 (qt, *J* = 5.0, 1.4 Hz, 1H), 4.42-4.38 (m, 1H), 4.33-4.28 (m, 0H), 1.99 (s, 1H), 1.93-1.73 (m, 2H), 0.90 (s, 9H), 0.11 (s,

6H) ppm; ^{13}C NMR (100 MHz, $CDCl_3$) δ 141.1, 113.9, 80.7, 70.6, 70.5, 69.1, 68.6, 68.4, 68.1, 41.9, 26.5, 25.7, 17.9, -4.6, -5.0 ppm; HRMS (ESI, M+Na) calcd. for $C_{14}H_{23}D_3O_2SiNa$ 280.1788, found 280.1804.

(7*S*)-2,2,3,3,9,9,10,10-octamethyl-5-(prop-2-yn-1-yl-1,1-*d*2)-7-vinyl-4,8-dioxa-3,9- disilaundecane-5-*d* (16).

To a solution of alkyne-d3 **(-)-15** (259.1 mg, 1.0 mmol) in CH_2Cl_2 (2.0 mL) was added imidazole (116.4 mg, 1.71 mmol), *tert*-

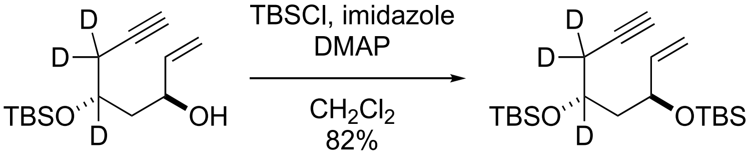

butyldimethylchlorosilane (167.3 mg, 1.11 mmol), 4-dimethyl amino pyridine (6.8 mg, 0.056 mmol) at room temperature, and the resulting mixture was stirred at room temperature for 1 h. The reaction mixture was quenched with H_2O (10 mL) and the mixture was extracted with a solution of dichloromethane three times. The combined organic layer was washed with brine, dried over $MgSO_4$, filtered, and concentrated *in vacuo*. The residue was purified by column chromatography on silica gel (*n*-hexane/ethyl acetate = 100:1) to give **16** (308.9 mg, 82%) as a colorless oil. Spectral data for **16**: $[\alpha]^{25}_D$= -7.1 (*c* 0.2, $CHCl_3$); ^{1}H NMR (400 MHz, $CDCl_3$) δ 5.87-5.76 (m, 1H), 5.20-5.02 (m, 2H), 4.23-4.19 (m, 1H), 1.97 (s, 1H), 1.91-1.78 (m, 1H), 1.65 (dd, *J* = 13.8, 4.8 Hz, 1H), 0.89 (s, 18H), 0.09-0.04 (m, 12H) ppm; ^{13}C NMR (100 MHz, $CDCl_3$) δ 141.9, 114.2, 81.3, 71.6, 70.1, 67.6, 67.4, 45.7, 27.6, 27.3, 25.9, 25.9, 18.2, 18.1, -3.7, -4.2, -4.4, -4.6 ppm; HRMS (ESI, M+Na) calcd. for $C_{20}H_{37}D_3O_3Si_2Na$ 394.2653, found 394.2677.

25(OH)D3-*d*3 (2-*d*3).

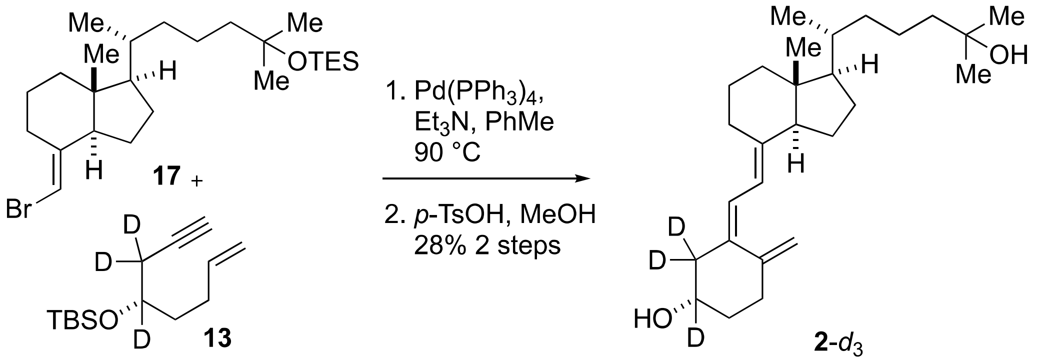

To a solution of bromoolefin **17** (38.6 mg, 0.082 mmol) and **13** (21.7 mg, 0.069 mmol) and Et_3N (0.5 mL) in toluene (0.5 mL) was added $Pd(PPh_3)_4$ (8.0 mg, 0.007 mmol) at room temperature, then the resulting mixture was heated at 90 °C. After stirring for 1 h, the reaction mixture was filtered through a pad of celite, and the filtrates were concentrated *in vacuo*. The residue was chromatographed on silica gel (*n*-hexane/ethyl acetate = 100:1) and PTLC (*n*-hexane) to give coupling product (16.0 mg). To a solution of the coupling product (16.0 mg, 0.0255 mmol) in MeOH (0.42 mL) was added p-TsOH (17.2 mg, 0.10 mmol) at 0 °C. After stirring for 0.5 h, the reaction was quenched with saturated $NaHCO_3$ aq., and the resulting mixture was extracted with ethyl acetate three times. The combined organic layer was washed with brine, dried over $MgSO_4$, filtered, and concentrated *in vacuo*. The residue was chromatographed on silica gel (*n*-hexane/ethyl acetate = 2:1) and PTLC (Chloroform) to give **2**-*d*3 (7.6 mg, 28% 2 steps). **2**-*d*3 was purified by reverse phase HPLC (MeOH/0.1% formic acid=63/37 as elutent with a flow rate of 1.0 mL min^{-1}, 8.6 min, 265 nm UV detection) to give **2**-*d*3 as a yellow oil. Spectral data for **2**-*d*3: $[\alpha]^{25}_D$= +41.4 (*c* 0.03, $CHCl_3$); ^{1}H NMR (400 MHz, $CDCl_3$) δ 6.23 (d, *J* = 11.5 Hz, 1H), 6.03 (d, *J* = 11.5 Hz, 1H), 5.05 (s, 1H), 4.82 (s, 1H), 2.82 (d, *J* = 12.6 Hz, 1H), 2.37-2.42 (m, 1H), 2.15-2.20 (m, 1H), 1.96-2.04 (m, 2H), 1.84-1.94 (m, 2H), 1.25-1.70 (m, 16H), 1.21 (s, 6H), 0.95 (d, *J* = 5.2 Hz, 3H), 0.54 (s, 3H) ppm; ^{13}C NMR (100 MHz, $CDCl_3$) δ 145.2, 142.4,

135.1, 122.5, 117.6, 112.5, 71.2, 56.6, 56.4, 46.0, 44.5, 40.6, 36.5, 36.2, 35.1, 32.0, 29.4, 29.3, 29.1, 27.8, 23.7, 22.3, 20.9, 18.9, 12.1 ppm; HRMS (ESI, M+Na) calcd. for $C_{27}H_{41}D_3O_2Na$ 426.3427, found 426.3454.

25(OH)D$_3$ 23,26-lactone-*d*$_3$ (6-*d*$_3$).

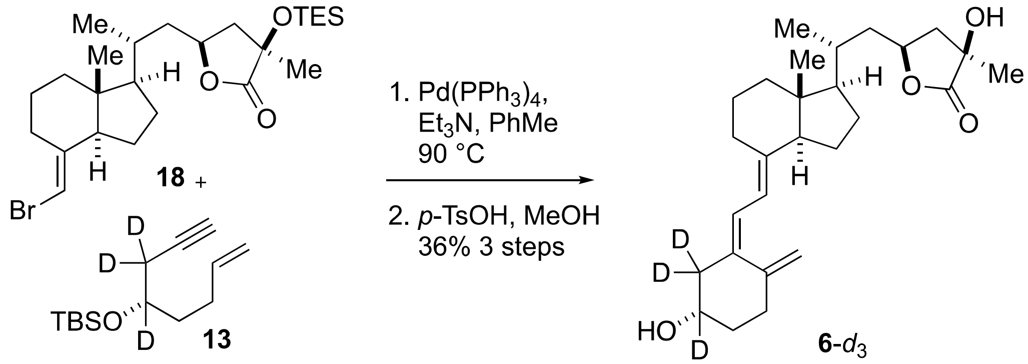

To a solution of (23*S*,25*R*)-bromoolefin **18** (26.5 mg, 0.053 mmol) and **13** (13.8 mg, 0.044 mmol) and Et_3N (0.3 mL) in toluene (0.3 mL) was added $Pd(PPh_3)_4$ (5.1 mg, 0.004 mmol) at room temperature, then the resulting mixture was heated at 90 °C. After stirring for 2 h, the reaction mixture was filtered through a pad of celite, and the filtrates were concentrated *in vacuo*. The residue was chromatographed on silica gel (*n*-hexane/ethyl acetate = 20:1) to give coupling product (21.7 mg). To a solution of the coupling product (21.7 mg, 0.033 mmol) in MeOH (0.3 mL) was added *p*-TsOH (22.4 mg, 0.13 mmol) at 0 °C. After stirring for 2 h, the reaction was quenched with saturated $NaHCO_3$ aq., and the resulting mixture was extracted with ethyl acetate three times. The combined organic layer was washed with brine, dried over $MgSO_4$, filtered, and concentrated *in vacuo*. The residue was chromatographed on silica gel (*n*-hexane/ethyl acetate = 1:1) and PTLC (*n*-hexane/ethyl acetate = 2:1) to give **6**-*d*$_3$ (5.1 mg, 36% 2 steps). **6**-*d*$_3$was purified by reverse phase HPLC (MeOH/0.1% formic acid=63/37 as elutent with a flow rate of 1.0 mL min^{-1}, 27.1 min, 265 nm UV detection) to give **6**-*d*$_3$ as a yellow oil. Spectral data for **6**-*d*$_3$: $[\alpha]^{25}{}_D$= +58.8 (*c* 0.02, $CHCl_3$);^{1}H NMR (400 MHz, $CDCl_3$) δ6.23 (d, *J* = 11.4 Hz, 1H), 6.03 (d, *J* = 10.5 Hz, 1H), 5.05 (s, 1H), 4.81 (s, 1H), 4.54-4.36 (m, 1H), 3.75-3.56 (m, 2H), 2.83 (d, *J* = 12.8 Hz, 1H), 2.51-2.31 (m, 2H), 2.26-2.10 (m, 1H), 2.11-1.80 (m, 6H), 1.81-1.43 (m, 8H), 1.42-1.15 (m, 6H), 1.02 (d, *J* = 6.4 Hz, 3H), 0.55 (s, 3H) ppm; ^{13}C NMR (100 MHz, $CDCl_3$) δ179.3, 145.0, 141.7, 135.3, 122.3, 117.7, 112.5, 73.3, 56.5, 56.2, 45.8, 43.2, 41.7, 40.4, 35.0, 33.8, 31.9, 29.7, 28.9, 27.9, 24.5, 23.4, 22.2, 19.3, 14.1, 12.0 ppm; HRMS (ESI, M+K) calcd. for $C_{27}H_{37}D_3O_4K$ 470.2752, found 470.2774.

1,25(OH)$_2$D$_3$ 23,26-lactone-*d*$_3$ (7-*d*$_3$).

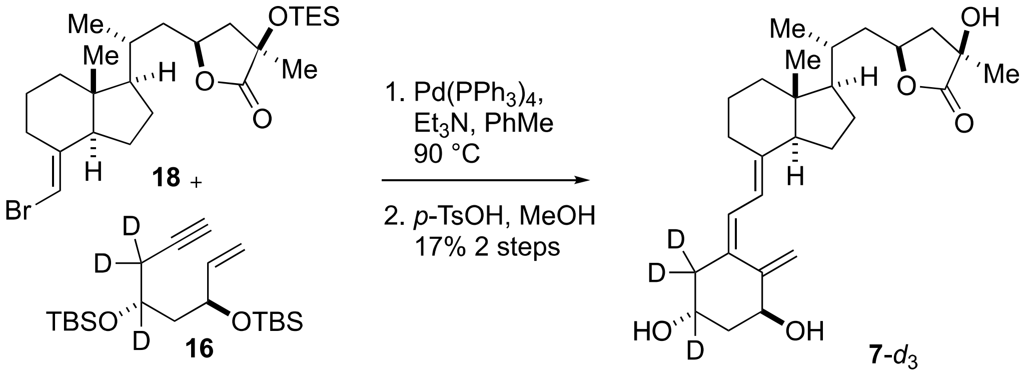

To a solution of (23*S*,25*R*)-bromo olefin **18** (38.6 mg, 0.082 mmol) and **16** (21.7 mg, 0.069 mmol) and Et_3N (0.5 mL) in toluene (0.5 mL) was added $Pd(PPh_3)_4$ (8.0 mg, 0.007 mmol) at room temperature, then the resulting mixture was heated at 90 °C. After stirring for 2 h, the reaction mixture was filtered through a pad of Celite, and the filtrates were concentrated *in vacuo*. The residue was chromatographed on silica gel (*n*-hexane/ethyl acetate = 100:1) to give coupling product (16.0 mg).

To a solution of the coupling product (16.0 mg, 0.025 mmol) in MeOH (0.4 mL) was added *p*-TsOH (17.2 mg, 0.1 mmol) at 0 °C. After stirring for 2 h, the reaction was quenched with saturated $NaHCO_3$ aq., and the resulting mixture was extracted with ethyl acetate three times. The combined organic layer was washed with brine, dried over $MgSO_4$, filtered, and concentrated *in vacuo*. The residue was chromatographed on silica gel (*n*-hexane/ethyl acetate = 4:1) to give **7**-d_3 (8.1 mg, 17% 2 steps) as a yellow oil. Spectral data for **7**-d_3: $[\alpha]^{25}_{D}$= +57.9 (*c* 0.04, $CHCl_3$); ^{1}H NMR (300 MHz, $CDCl_3$) δ 6.23 (d, J = 11.0 Hz, 1H), 6.02 (d, J = 10.7 Hz, 1H), 5.18 (s, 1H), 4.86 (s, 1H), 4.39-4.30 (m, 3H), 2.83 (d, J = 11.4 Hz, 1H), 2.35 (dd, J = 12.6, 5.3 Hz, 1H), 1.98 (d, J = 9.6 Hz, 3H), 1.66 (d, J = 5.8 Hz, 4H), 1.56-1.25 (m, 12H), 0.99 (d, J = 4.1 Hz, 3H), 0.54 (s, 3H) ppm; ^{13}C NMR (100 MHz, $CDCl_3$) δ 179.46, 147.56, 142.65, 133.03, 124.85, 117.24, 111.85, 73.34, 70.81, 60.41, 56.45, 56.21, 45.86, 43.25, 42.63, 41.66, 40.38, 33.81, 29.68, 28.97, 27.92, 24.46, 23.47, 22.20, 21.23, 21.05, 19.26, 17.42, 14.18, 11.95 ppm; HRMS (ESI, M+Na) calcd. for $C_{27}H_{37}D_3O_5Na$ 470.2962, found 470.2963.

2. Experimental procedure for LC-MS/MS analysis using the isotope dilution method

2.1 Materials

Standard compounds: 25OHD3, a component of the JeoQuant^{TM} for LC-MS/MS analysis of vitamin D metabolites, was obtained from JEOL (Akishima, Tokyo, Japan). 25OHD3-23,26-Lactone (**6**) and 1,25(OH)2D3-23,26-lactone (**7**) were synthesized by our group [35].

Internal standards: 25OHD3-*d*3 (**2**-*d*3), 25OHD3-23,26-lactone-*d*3 (**6**-*d*3), 1,25(OH)2D3-23,26-lactone-*d*3 (**7**-*d*3) were synthesized as shown in scheme 4.

Derivatization reagent: DAP-PA ethyl acetate solution (0.5 mg/mL) was a component of the JeoQuant^{TM} (JEOL, Akishima, Tokyo, Japan).

Human serum: Pooled human serum was obtained from Cosmo Bio (Tokyo, Japan).

2.2 Preparation of standard samples (DAP-PA-derivatized vitamin D metabolites)

DAP-PA derivatization: A solution of DAP-PA in ethyl acetate (100 µL, excess) was preheated at 80 °C for 15 min, then added to the dried samples (D3 derivatives) at room temperature. The mixtures were allowed to stand for 15 min (Scheme 4), then the solvent was removed in a centrifugal evaporator. The residue was dissolved in aqueous acetonitrile (50%, 50 µL).

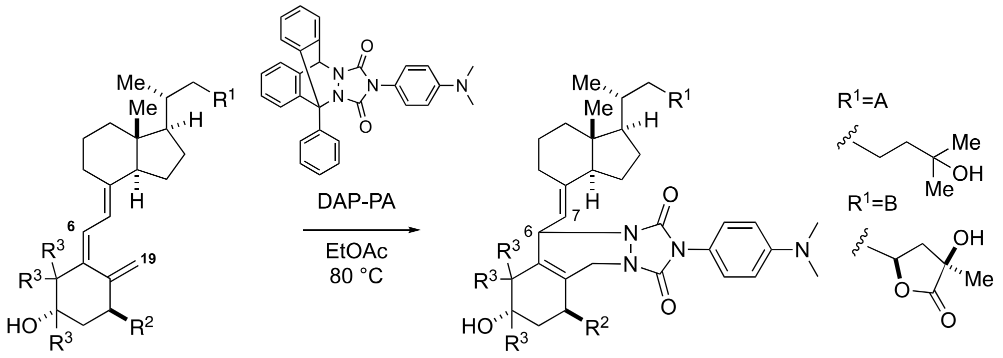

R1=A, R2,R3=H: **2** R3=D: **2**-*d*3
R1=B, R2,R3=H: **6** R3=D: **6**-*d*3
R1=B, R2=OH,R3=H:**7** R3=D: **7**-*d*3

R1=A, R2,R3=H: **2**-DAP R3=D: **2**-*d*3-DAP
R1=B, R2,R3=H: **6**-DAP R3=D: **6**-*d*3-DAP
R1=B, R2=OH,R3=H: **7**-DAP R3=D: **7**-*d*3-DAP

Scheme S1. Diels-Alder reaction of DAP-PA with D3 derivatives

2.3 Optimization of LC MS/MS conditions

The quantification of D3 metabolites in serum was carried out by LC-MS/MS analysis of the DAP-PA derivatives. The analytical procedures were as follows.

LC-MS/MS analyses: LC-MS/MS analyses were performed using a Waters Xevo TQ-XS triple quadrupole mass spectrometer (Milford, MA, USA) equipped with a Waters ACQUITY UPLC I-Class liquid chromatography system (Milford, MA, USA) or a Shimadzu LCMS-8040 triple quadrupole mass spectrometer (Kyoto, Japan) equipped with a Shimadzu UFLC LC-20AD liquid chromatography system (Kyoto, Japan). A binary gradient system with 0.1% formic acid (A) and 0.1% formic acid acetonitrile (B) was employed. Samples were dissolved in 50 µL of 50% aqueous acetonitrile and loaded onto a CAPCELL CORE C18 (2.7 µm) 2.1 mm I.D. x 75 or 100 mm column (Osaka Soda, Osaka, Japan) equilibrated with 0.1% formic acid 30% acetonitrile at a flow rate at 0.4 (25(OH)D3-23,26-lactone and 1,25(OH)2D3-23,26-lactone) or 0.3 (25(OH)D3) mL/min. The injection volume was 20 µL. The elution program for analyses of 25(OH)D3-23,26-lactone and 1,25(OH)2D3-23,26-lactone was as follows: 0→2.0 min, 30→45% B; 2.0→3.0 min, 45% B; 3.0→3.5 min, 45→52% B; 3.5→4.0 min, 52→90%B; 4.0→4.01 min, 90% B; 4.01→4.7 min, 30% B; 4.7→6.0 min, 30% B. For analysis of 25(OH)D3 the elution program was as follows: 0→0.5 min, 30→60% B; 0.5→3.5 min, 60% B; 3.5→3.51 min, 60→90% B; 3.51→5.0 min, 90% B; 5.0→5.01

min, 90→30% B; 5.01→6.5 min, 30% B. Ionization was done in the positive ion mode, and SRM was used for quantification. The transition (*m/z*) and collision energy (CE) values for the VD metabolites are summarized in Table 1. Operation and quantification analyses were done using Mass Lynx ver. 4.1 and Target Lynx Xs quantitative software (Waters) or LabSolutions ver. 5.89 software (Shimadzu), respectively.

2.4 Preparation of calibrators and internal standard solutions:

The JeoQuant™ calibrator contains not only $25OHD_3$ but also 3-epi-$25OHD_3$ at concentrations of 0.873 and 0.0940 ng/mL for calibrator level 1; 8.80, and 0.938 ng/mL for calibrator level 2; 44.0, and 4.69 ng/mL for calibrator level 3; 88.0, and 9.37 ng/mL for calibrator level 4, respectively. The calibrator for $25OHD_3$-23,26-lactone, $1,25(OH)_2D_3$-23,26-lactone, were obtained by means of two-fold dilutions of stock solution, and the concentration was 12.5 pg/mL for calibrator level 1; 25.0 pg/mL for calibrator level 2; 50.0 ng/mL for calibrator level 3; 100 ng/mL for calibrator level 4. Solutions of $25(OH)D_3$-d_3 (**2**-d_3), $25(OH)D_3$-23,26-lactone-d_3 (**6**-d_3), and $1,25(OH)_2D_3$-23,26-lactone-d_3 (**7**-d_3) were prepared at 200 pg/mL in 30% aqueous acetonitrile.

3. ^{1}H and ^{13}C NMR spectra

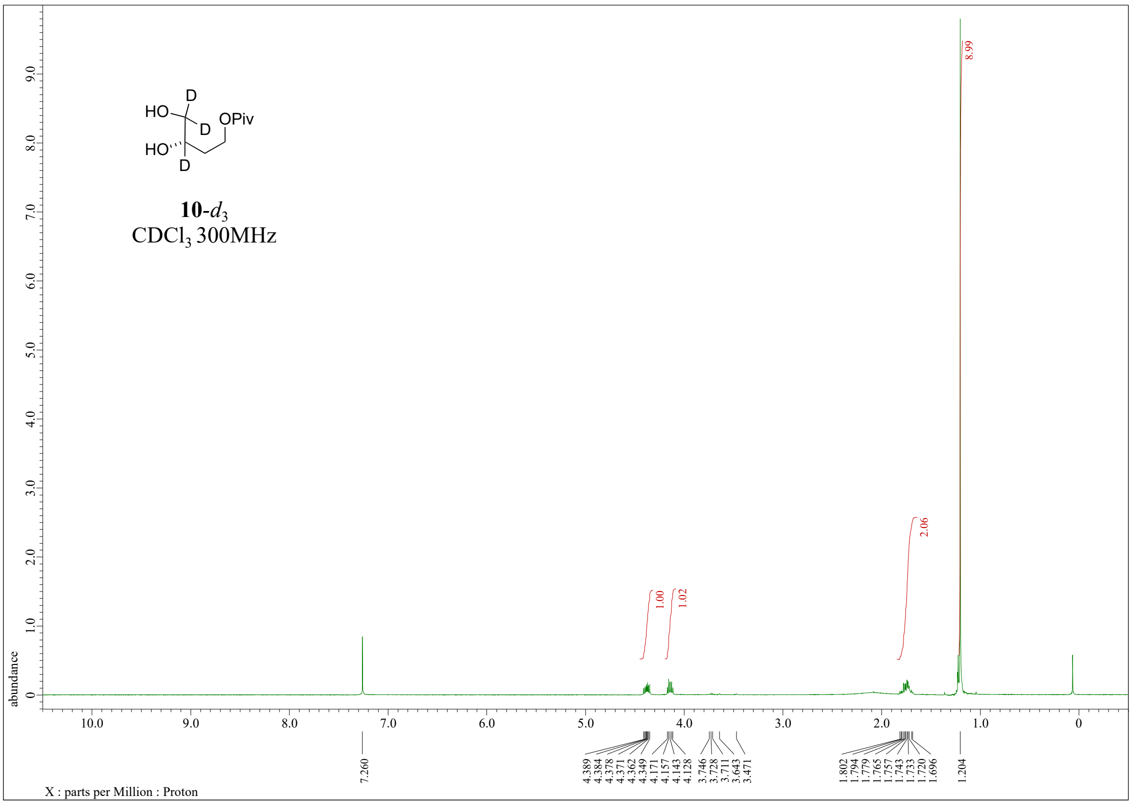

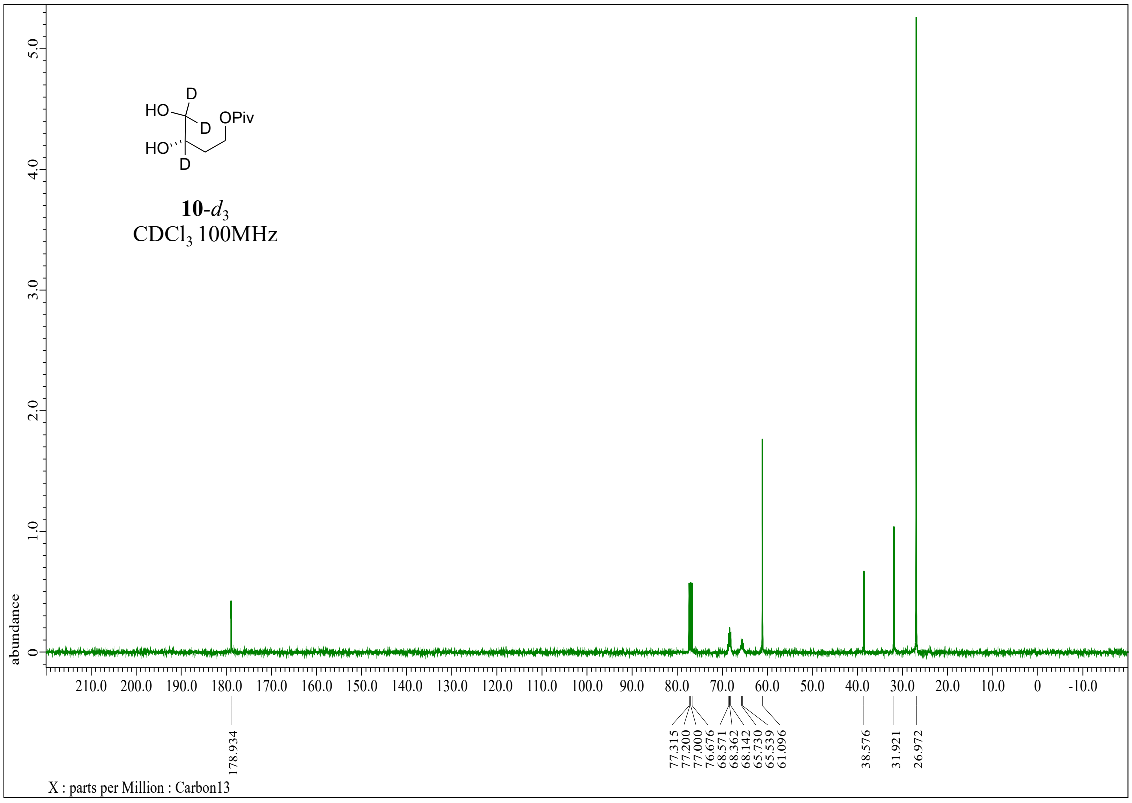

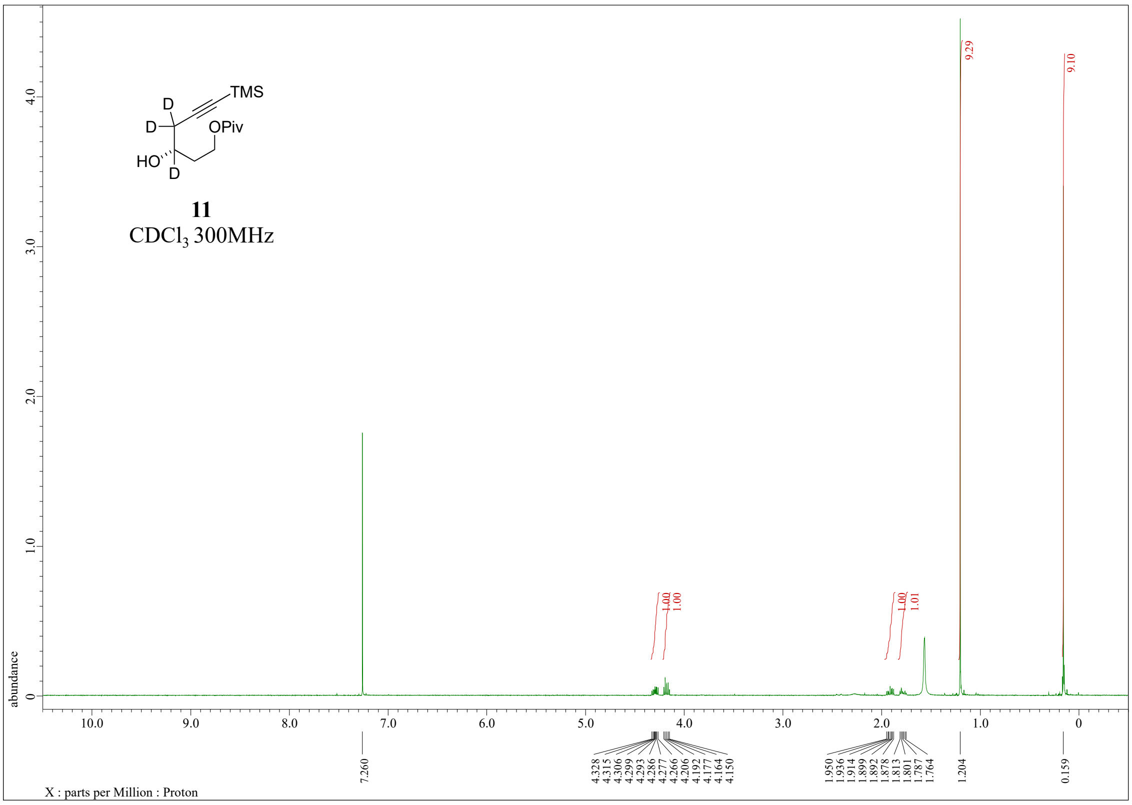
TMS
D
D
OPiv
HO
D
11
CDCl3 300MHz
abundance
X : parts per Million : Proton
7.260
1.204
0.159
9.29
9.10

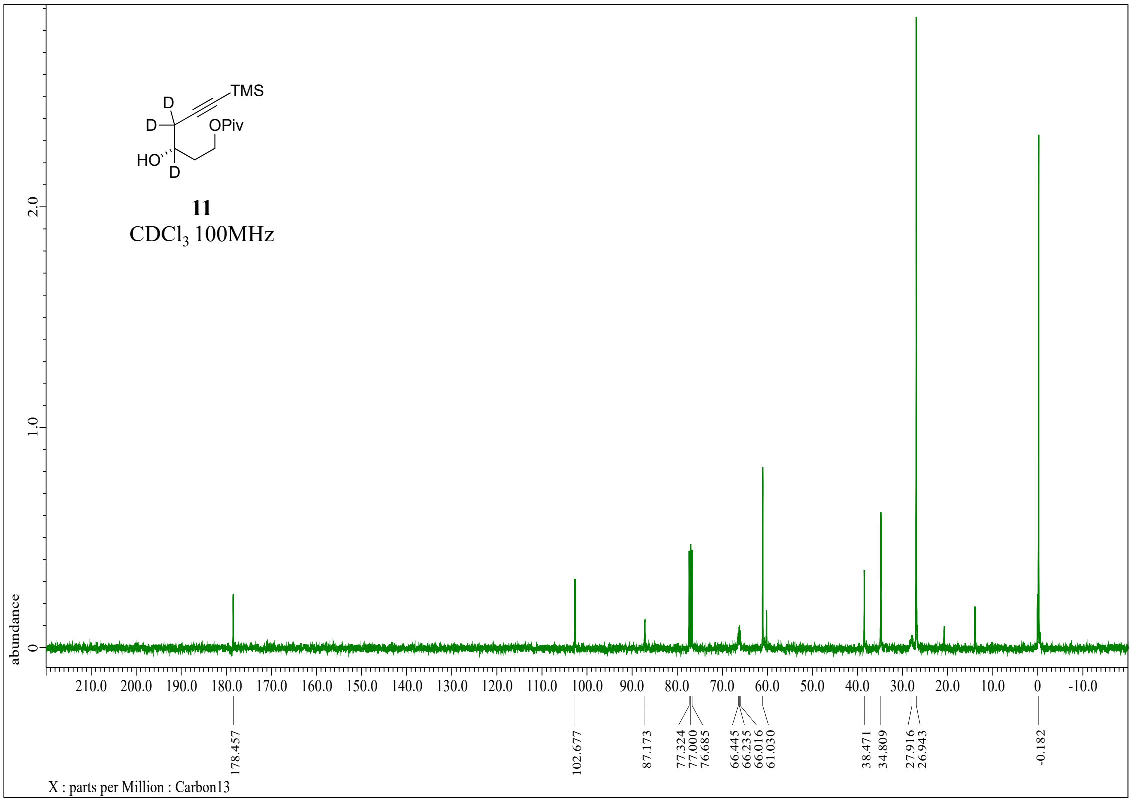
TMS
D
D
OPiv
HO
D
11
CDCl3 100MHz
abundance
X : parts per Million : Carbon13
178.457
102.677
87.173
77.324
77.000
76.685
66.445
66.235
66.016
61.030
38.471
34.809
27.916
26.943
-0.182

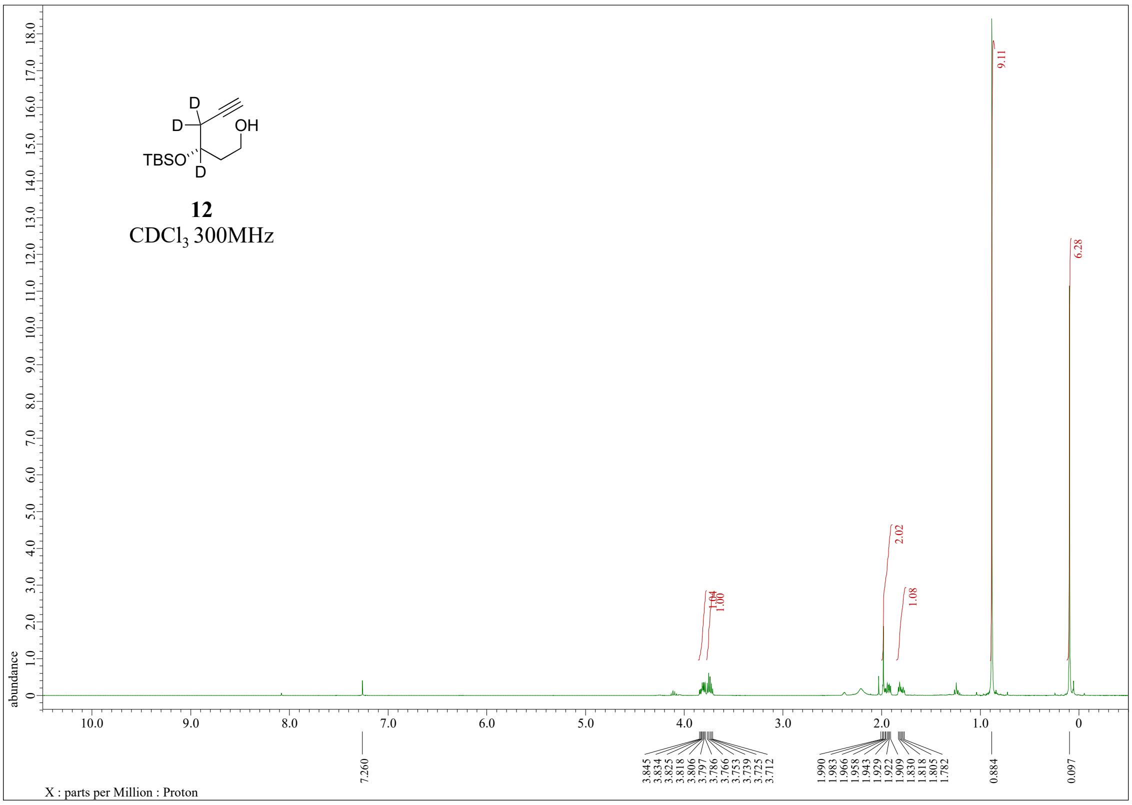
D
D
OH
TBSO
D
12
CDCl3 300MHz
abundance
9.11
6.28
2.02
1.08
1.04
1.00
7.260
0.884
0.097
X : parts per Million : Proton

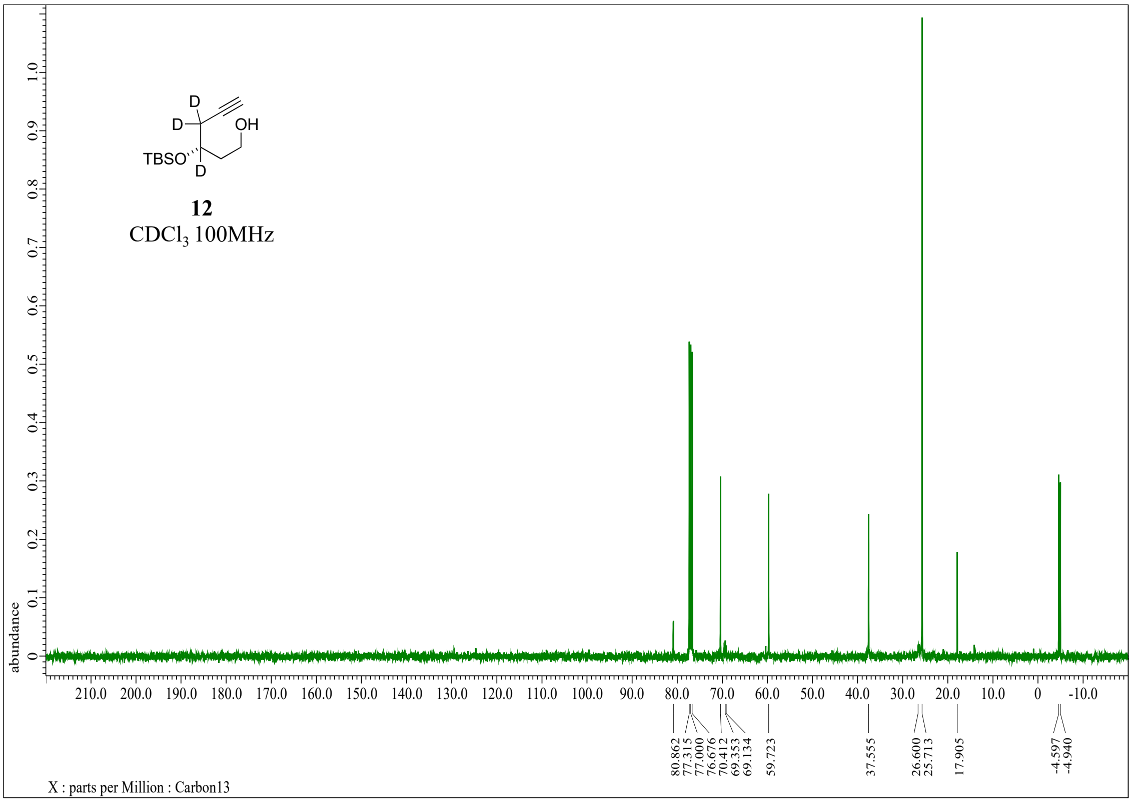
D
D
OH
TBSO
D
12
CDCl3 100MHz
abundance
80.862
77.315
77.000
76.676
70.412
69.353
69.134
59.723
37.555
26.600
25.713
17.905
-4.597
-4.940
X : parts per Million : Carbon13

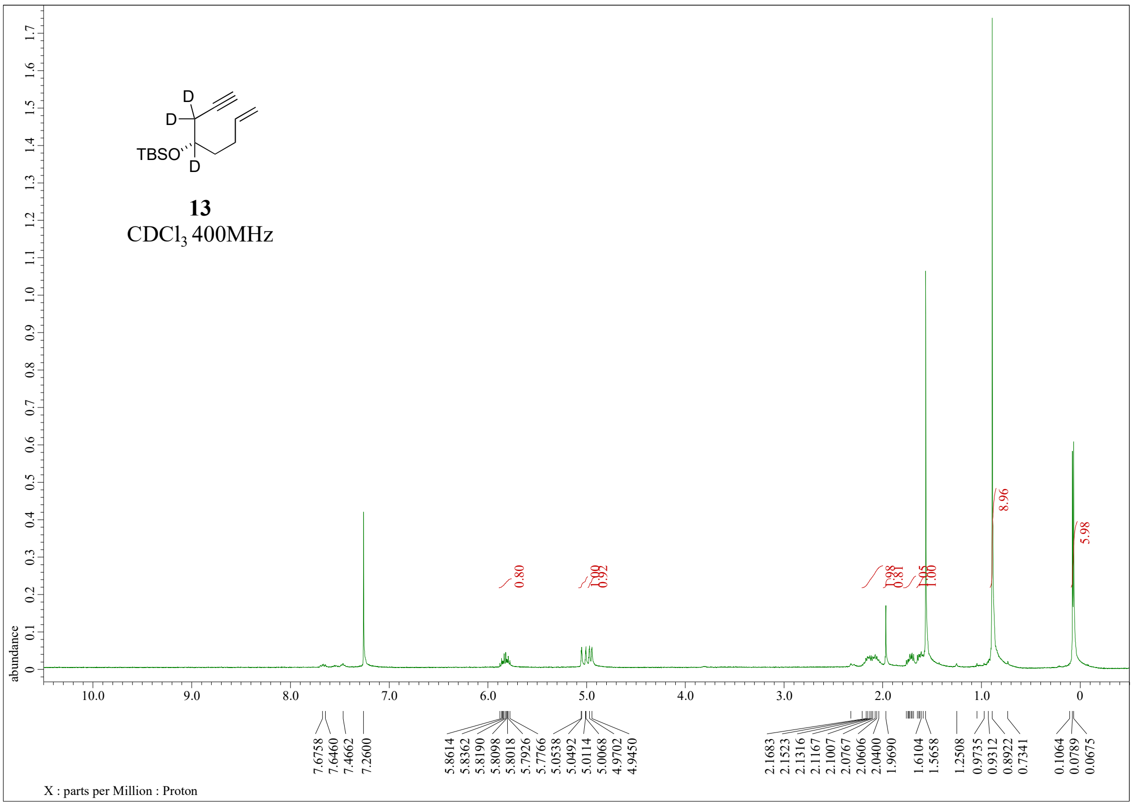
13
CDCl3 400MHz
TBSO
D
X : parts per Million : Proton
abundance

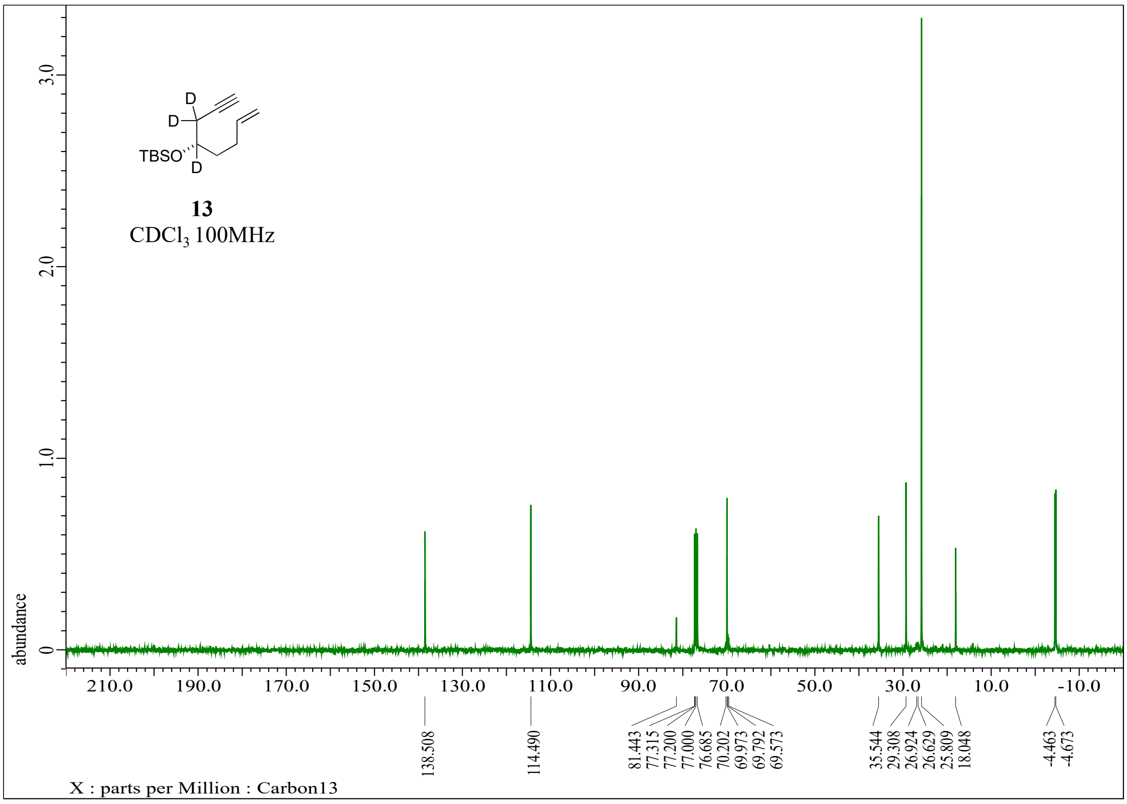
13
CDCl3 100MHz
TBSO
D
X : parts per Million : Carbon13
abundance

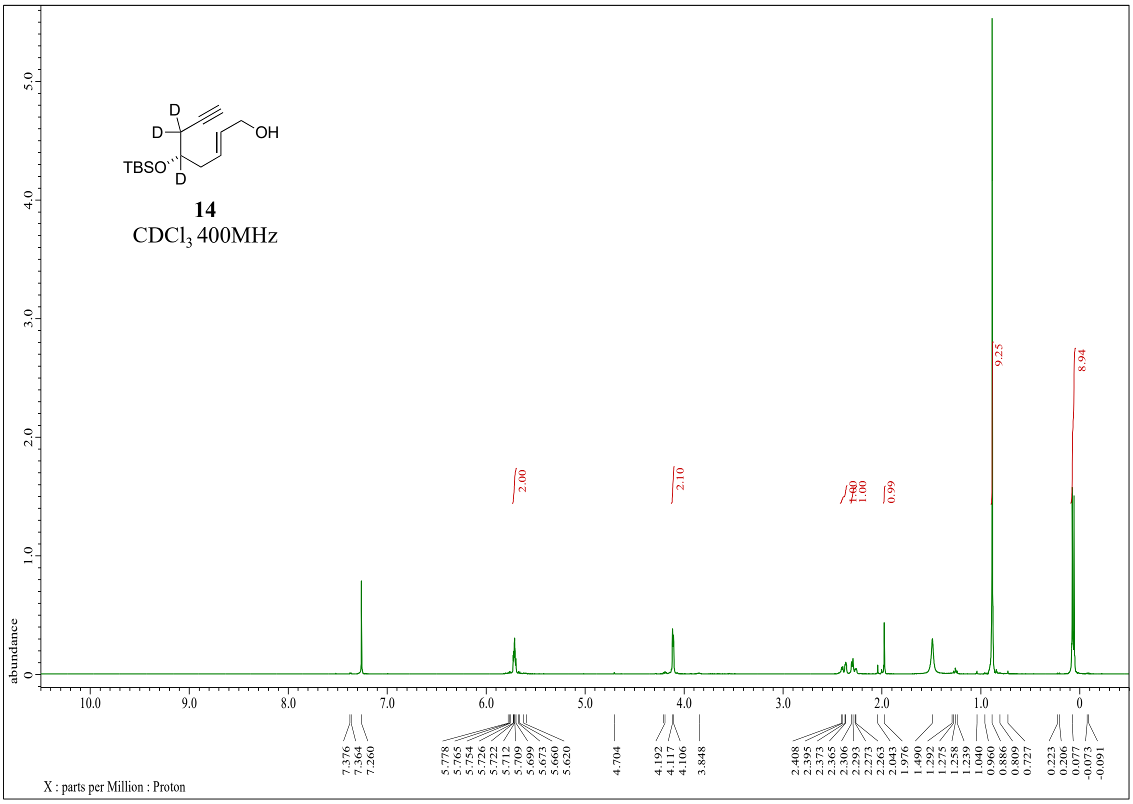
14
CDCl3 400MHz
TBSO
OH
X : parts per Million : Proton
abundance
2.00
2.10
1.00
0.99
9.25
8.94

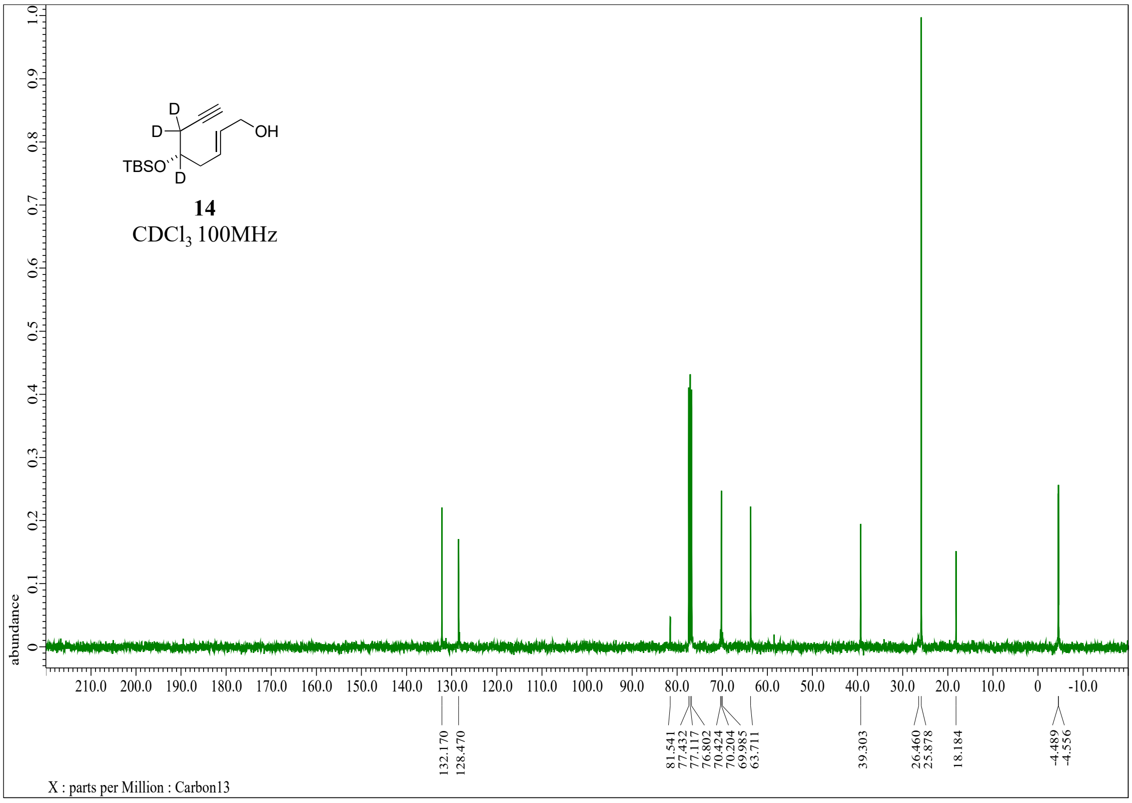
14
CDCl3 100MHz
TBSO
OH
X : parts per Million : Carbon13
abundance
132.170
128.470
81.541
77.432
77.117
76.802
70.424
70.204
69.985
63.711
39.303
26.460
25.878
18.184
-4.489
-4.556

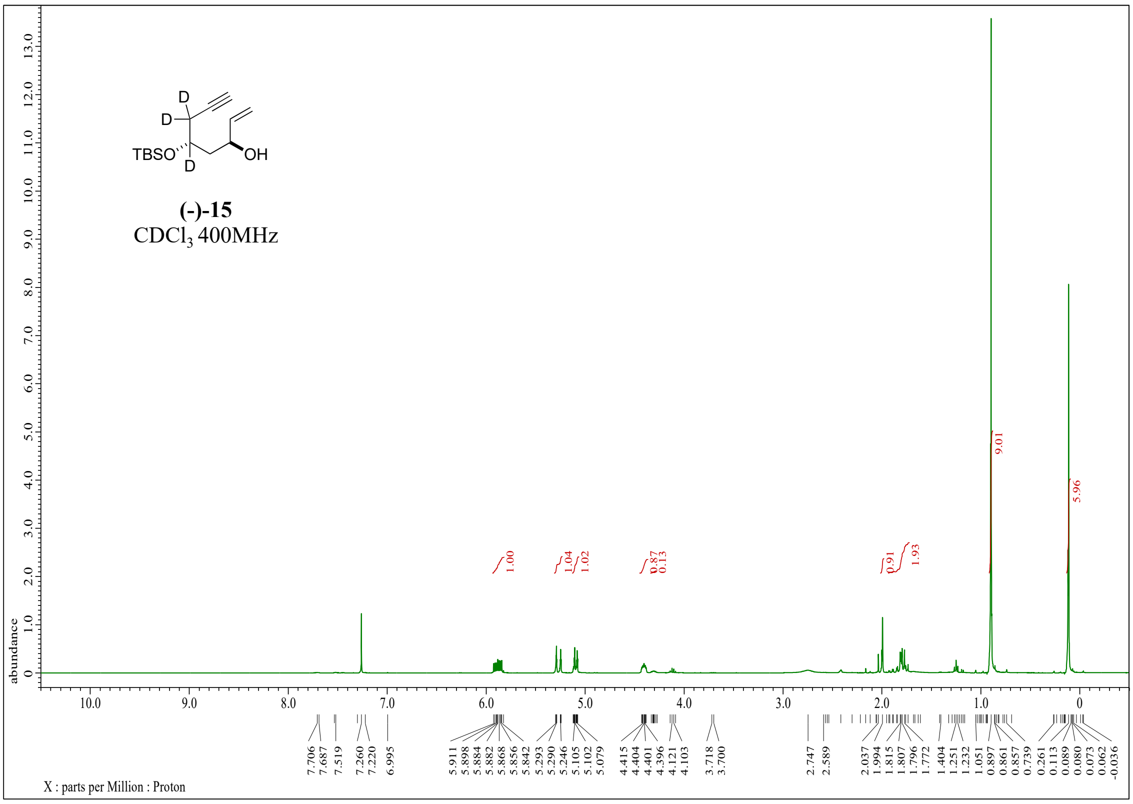
(-)-15
CDCl3 400MHz
abundance
X : parts per Million : Proton

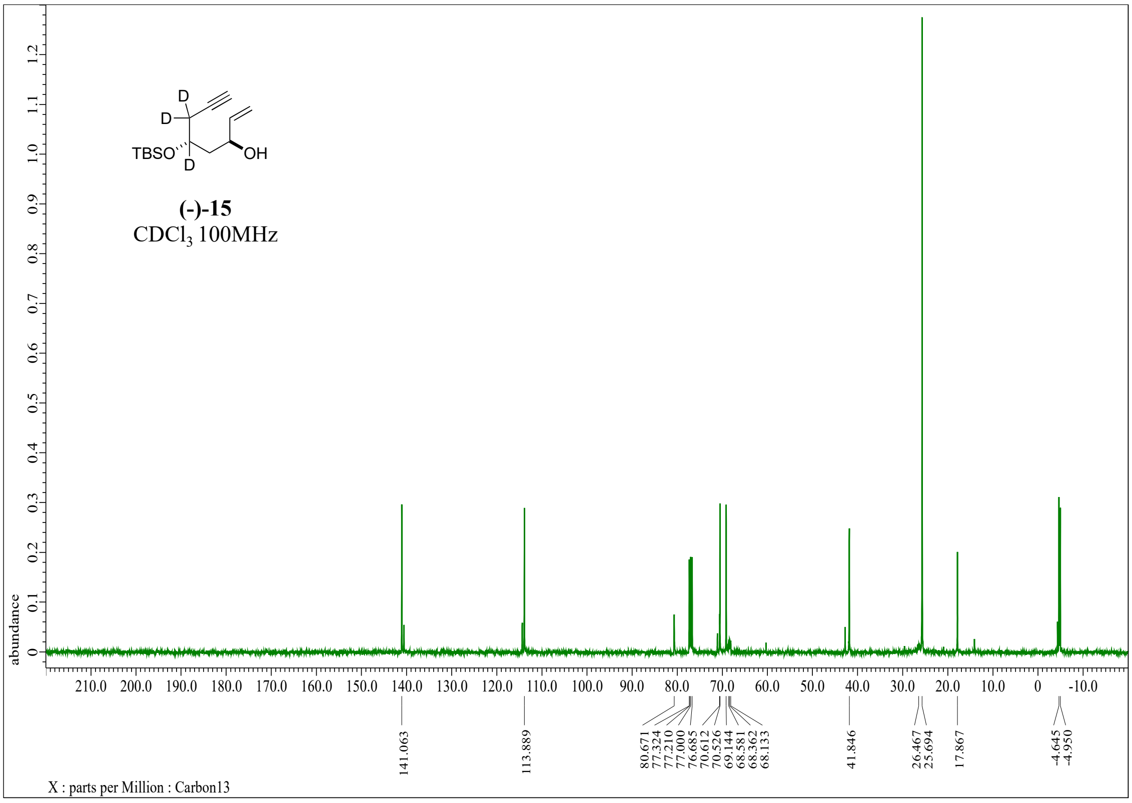
(-)-15
CDCl3 100MHz
abundance
X : parts per Million : Carbon13

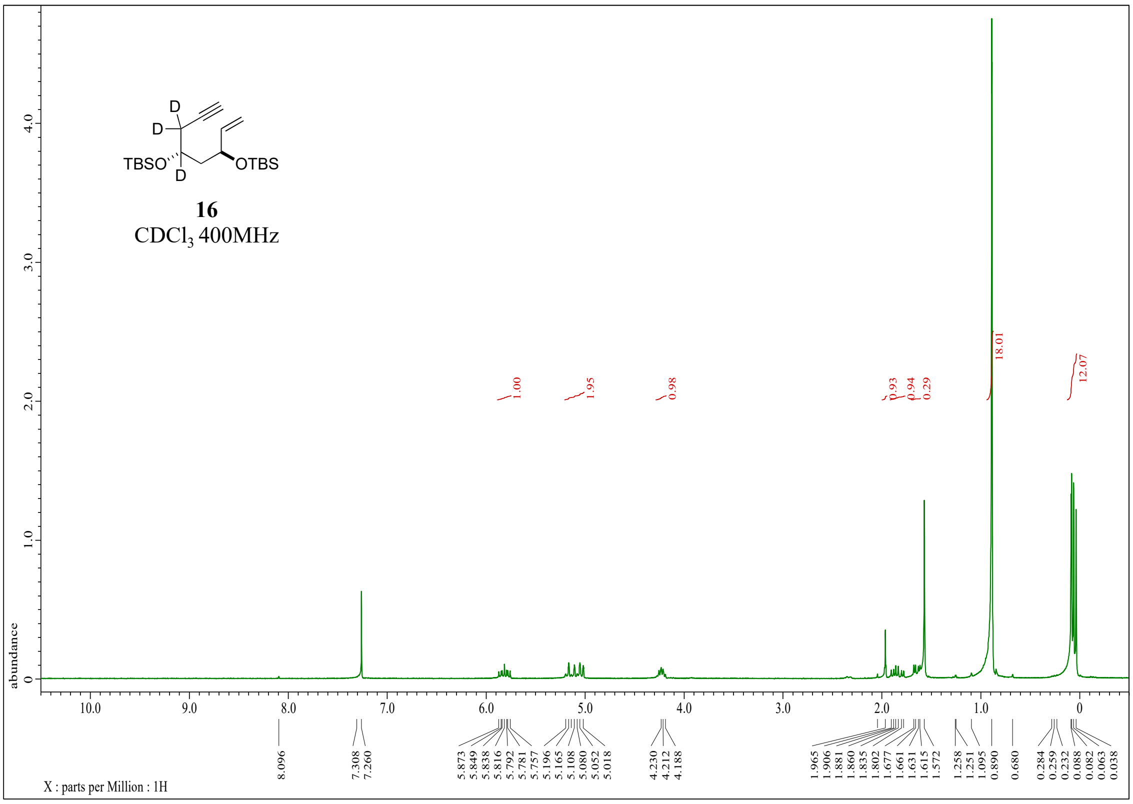

D
D
TBSO
D
OTBS
16
CDCl3 400MHz
1.00
1.95
0.98
0.93
0.94
0.29
18.01
12.07
abundance
X : parts per Million : 1H

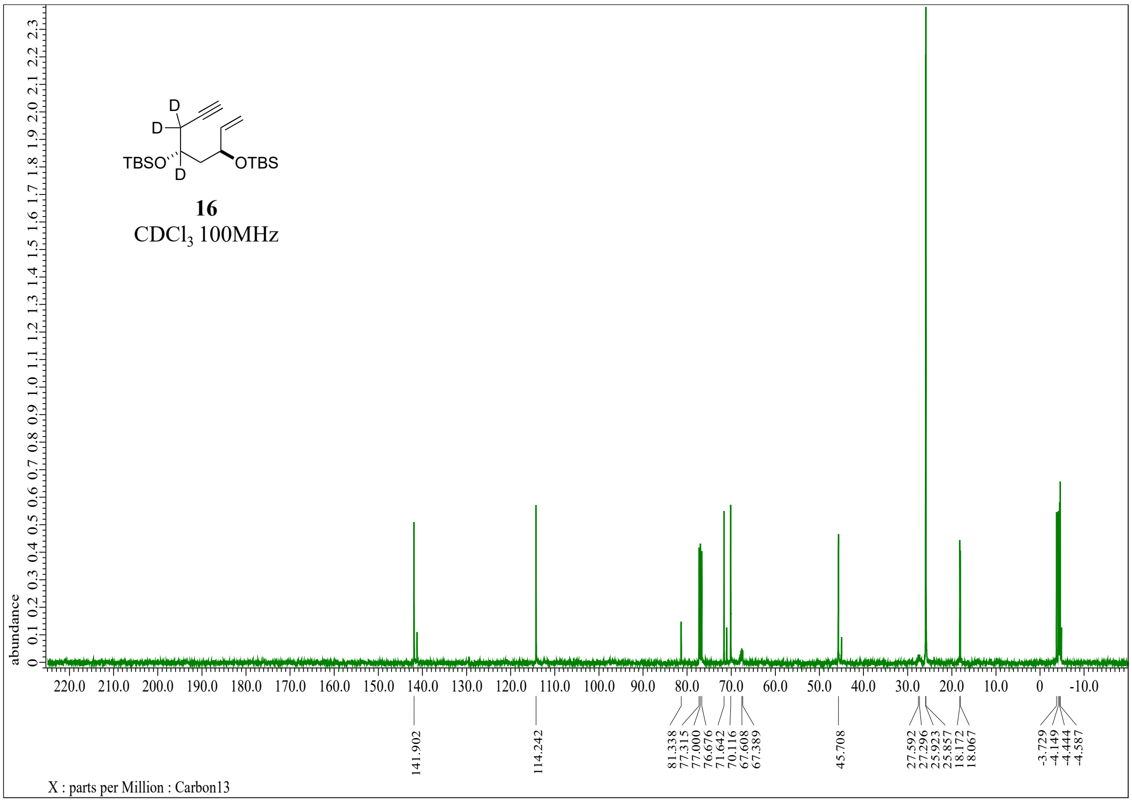

D
D
TBSO
D
OTBS
16
CDCl3 100MHz
141.902
114.242
81.338
77.315
77.000
76.676
71.642
70.116
67.608
67.389
45.708
27.592
27.296
25.923
25.857
18.172
18.067
-3.729
-4.149
-4.444
-4.587
abundance
X : parts per Million : Carbon13

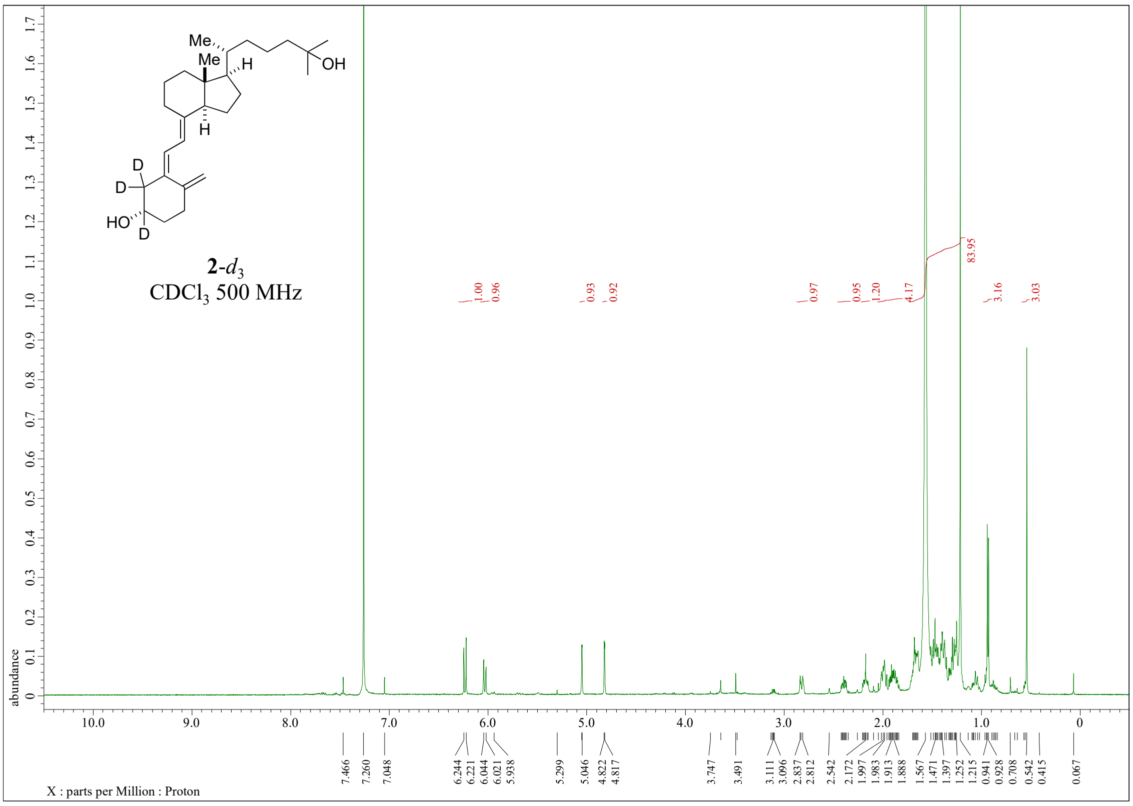
Me
Me
H
OH
H
D
D
HO
D
2-d3
CDCl3 500 MHz
abundance
X : parts per Million : Proton

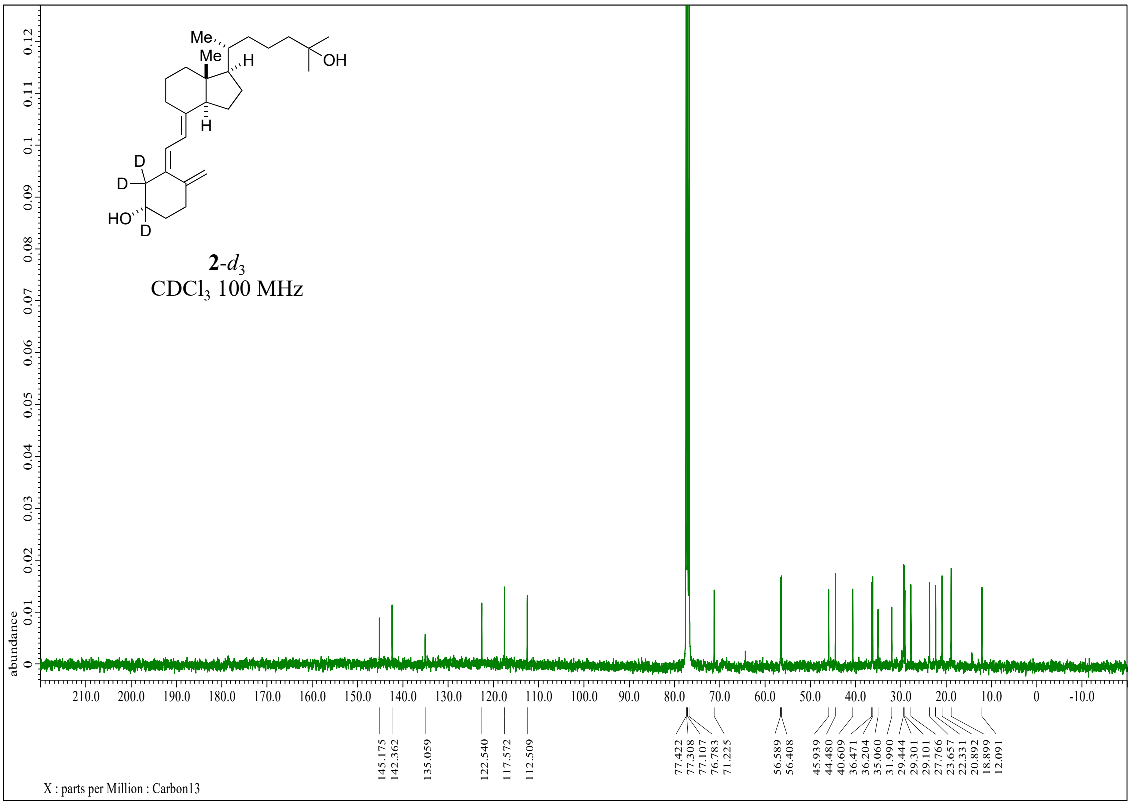
Me
Me
H
OH
H
D
D
HO
D
2-d3
CDCl3 100 MHz
abundance
X : parts per Million : Carbon13

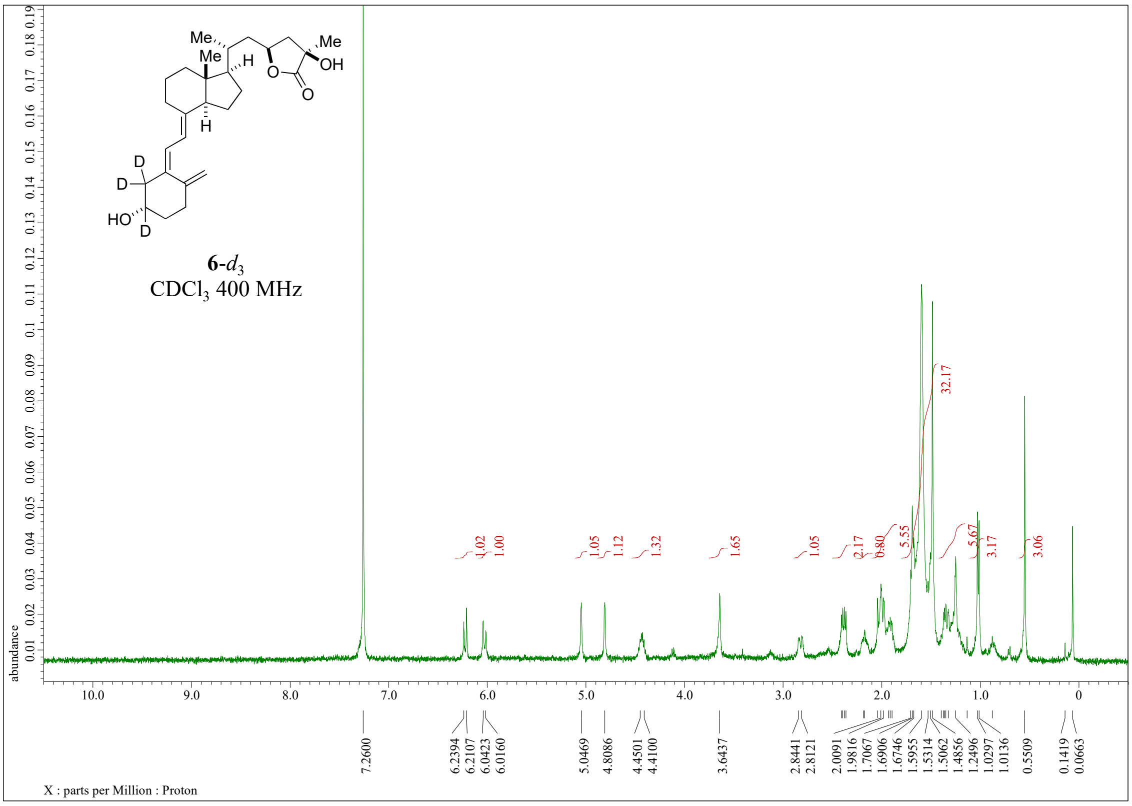

Me
Me
H
Me
OH
O
O
H
D
D
HO
D
6-d_3
CDCl3 400 MHz
abundance
X : parts per Million : Proton

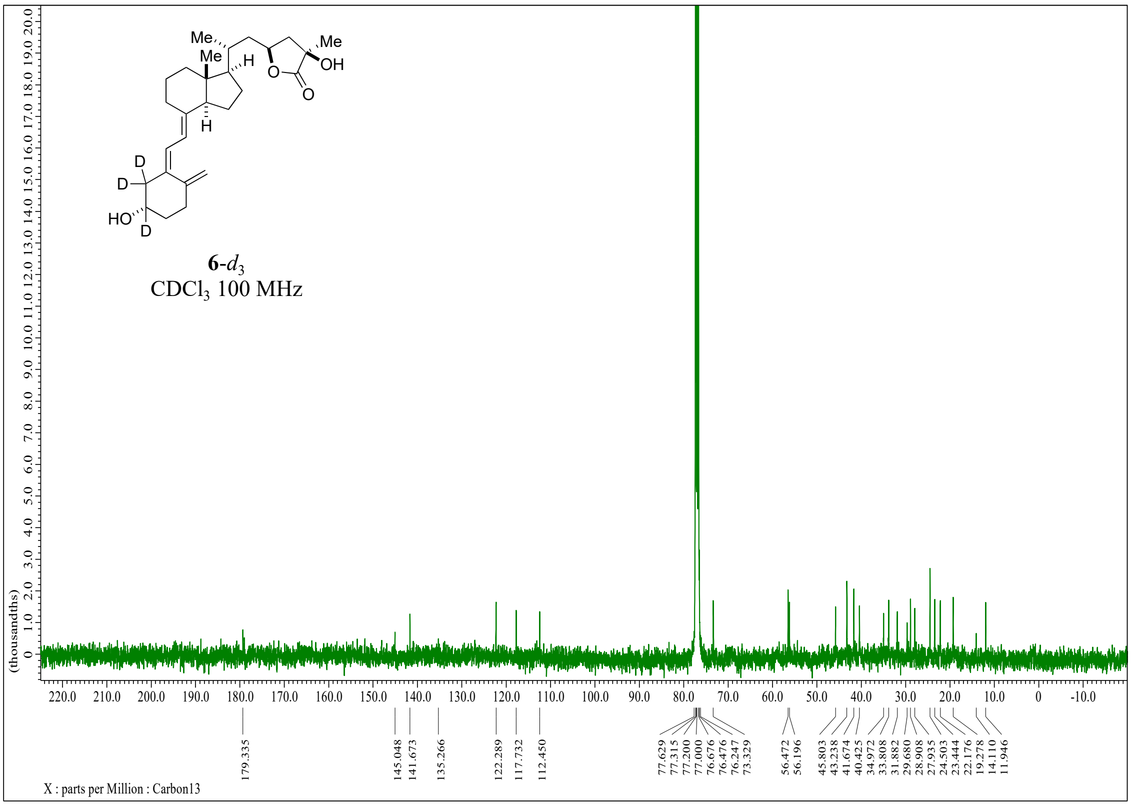

Me
Me
H
Me
OH
O
O
H
D
D
HO
D
6-d_3
CDCl3 100 MHz
(thousandths)
X : parts per Million : Carbon13

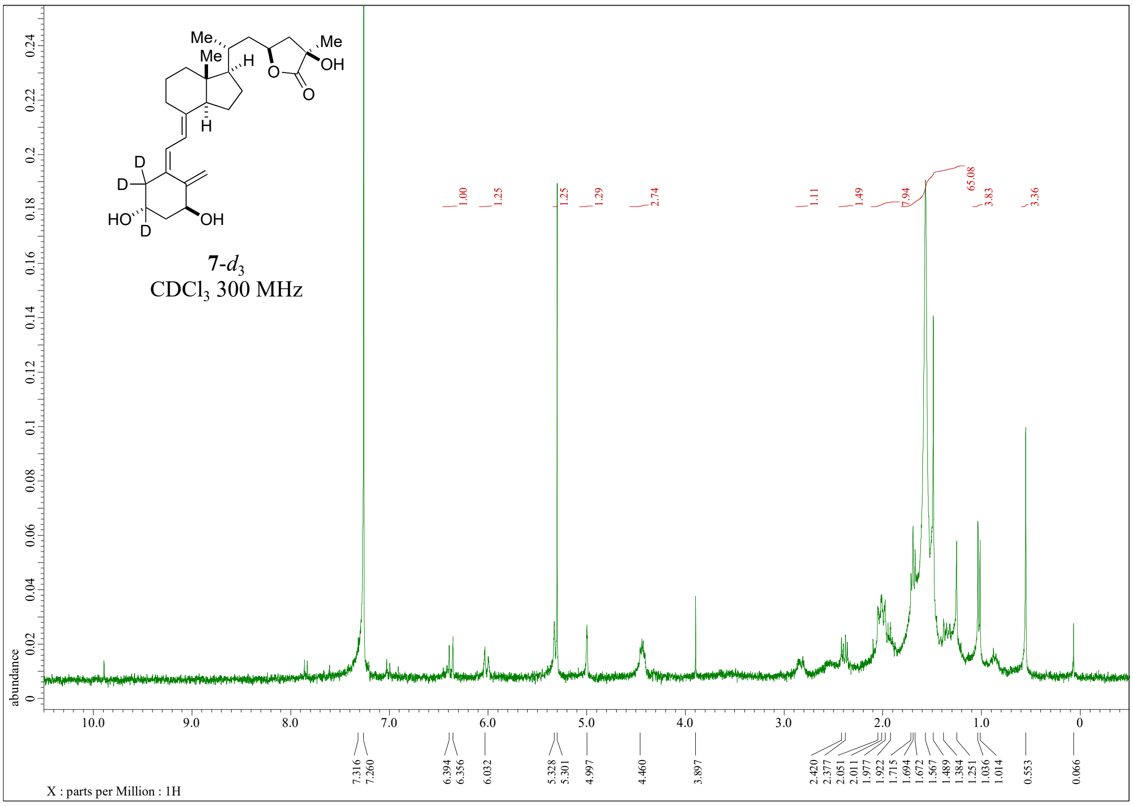
Me
Me
H
Me
OH
O
O
H
D
D
HO
D
OH
7-d_3
$CDCl_3$ 300 MHz
abundance
1.00
1.25
1.25
1.29
2.74
1.11
1.49
7.94
65.08
3.83
3.36
7.316
7.260
6.394
6.356
6.032
5.328
5.301
4.997
4.460
3.897
2.420
2.377
2.051
2.011
1.977
1.922
1.715
1.694
1.672
1.567
1.489
1.384
1.251
1.036
1.014
0.553
0.066
X : parts per Million : 1H

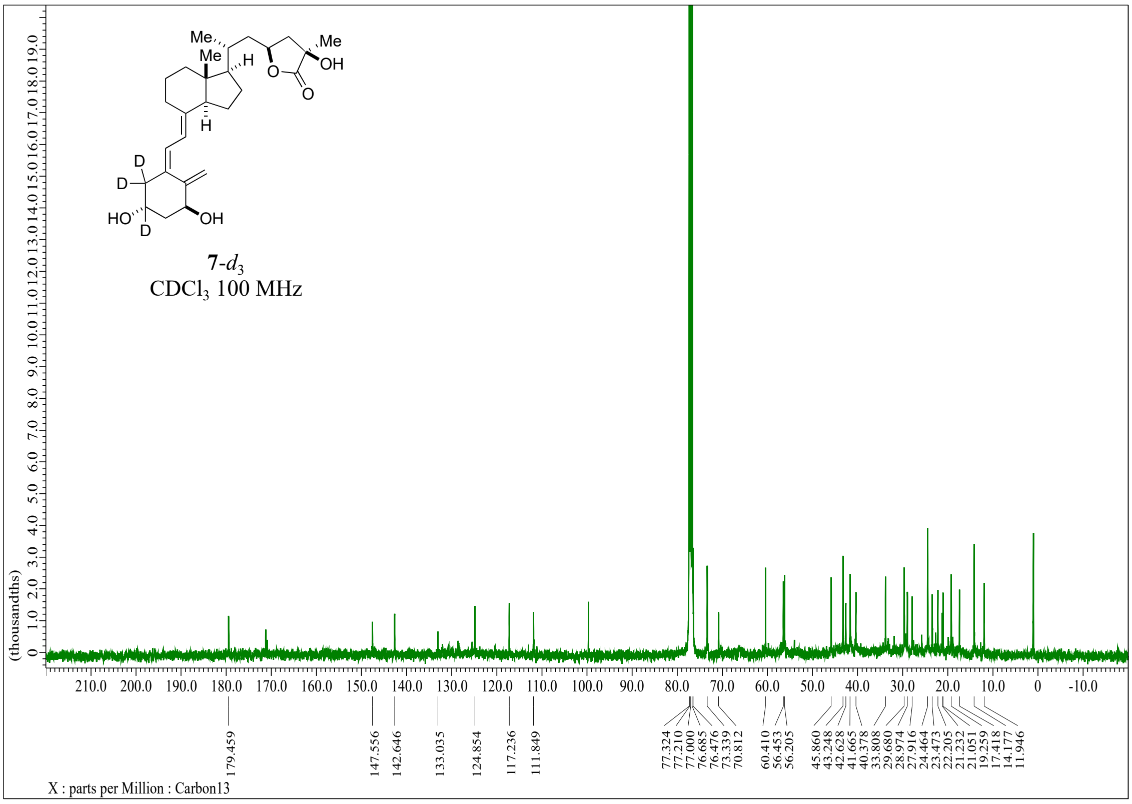
Me
Me
H
Me
OH
O
O
H
D
D
HO
D
OH
7-d_3
$CDCl_3$ 100 MHz
(thousandths)
179.459
147.556
142.646
133.035
124.854
117.236
111.849
77.324
77.210
77.000
76.685
76.476
73.339
70.812
60.410
56.453
56.205
45.860
43.248
42.628
41.665
40.378
33.808
29.680
28.974
27.916
24.464
23.473
22.205
21.232
21.051
19.259
17.418
14.177
11.946
X : parts per Million : Carbon13
